# Magnetic Resonance Spectroscopy of γ-Aminobutyric Acid and Glutamate Concentrations in Children With Attention-Deficit/Hyperactivity Disorder

**DOI:** 10.1001/jamanetworkopen.2020.20973

**Published:** 2020-10-16

**Authors:** Tasmia Hai, Rose Swansburg, Cynthia K. Kahl, Hannah Frank, Jean-François Lemay, Frank P. MacMaster

**Affiliations:** 1 Werklund School of Education, University of Calgary, Calgary, Alberta, Canada; 2 Department of Educational Psychology, University of Alberta, Edmonton, Alberta, Canada; 3 Department of Psychiatry, Cumming School of Medicine, University of Calgary, Calgary, Alberta, Canada; 4 Department of Psychology, Mount Royal University, Calgary, Alberta, Canada; 5 Department of Pediatrics, Cumming School of Medicine, University of Calgary, Calgary, Alberta, Canada

## Abstract

This cohort study assesses concentrations of γ-aminobutyric acid (GABA) and glutamate in children with attention-deficit/hyperactivity disorder (ADHD) compared with typically developing children.

## Introduction

Studies suggest that dysregulation of excitatory and inhibitory systems plays a role in attention-deficit/hyperactivity disorder (ADHD).^1^ However, few studies have examined γ-aminobutyric acid (GABA) dysfunction in children with ADHD.^2,3^ Furthermore, impulsivity and response inhibition have been associated with GABA.^4^ Proton magnetic resonance spectroscopy can quantify GABA and glutamate concentrations. To date, no study has investigated both glutamate and GABA concentrations in the anterior cingulate cortex (ACC) in children with ADHD. We hypothesized that there are greater concentrations of glutamate and of glutamine and glutamate (Glx) and a lower concentration of GABA in children with ADHD compared with typically developing control (TDC) participants.^1^ We also hypothesized that the longer GABA acquisition is feasible in unmedicated children with ADHD.

## Methods

This cohort study was approved by the University of Calgary Conjoint Health Research Ethics Board. Participants provided written assent, and parents or caregivers provided written informed consent. We enrolled 26 children with ADHD and 25 TDC participants between the ages of 7 and 18 years from May to December of 2019. To confirm eligibility as either a child with ADHD or a TDC participant, participants completed a structured diagnostic interview using the Mini-International Neuropsychiatric Interview for Children and Adolescents and a cognitive screener (the fifth edition of the Weschsler Intelligence Scale for Children) to establish no intellectual disability (standard score >80). Parents or cargivers also completed the third edition of Conners manual that assesses cognitive, behavioral, and emotions problems to support the presence or absence of ADHD. Participants with ADHD received a diagnosis by a health care professional, verified by an experienced developmental pediatrician (J.-F. L.), and underwent a 48-hour stimulant washout. All events occurred at the Alberta Children’s Hospital in Calgary, Alberta, Canada.

Magnetic resonance imaging was performed using a 32-channel head coil and a 3-T GE 750w scanner (General Electric). A T1-weighted acquisition was used for voxel placement (0.8-mm^3^ isotropic voxels). For proton magnetic resonance spectroscopy, a short-echo point-resolved spectroscopy (PRESS) acquisition (repetition time, 1.8 seconds; echo time, 30 milliseconds; 64 averages) for glutamate and Glx and a Mescher-Garwood (MEGA)–PRESS acquisition for GABA (repetition time, 1.8 seconds; echo time, 68 milliseconds; 256 averages) were applied to an angulated bilateral ACC voxel (30 × 20 × 40 mm; Figure). The magnetic resonance spectroscopy data were analyzed objectively using a linear combination model and Gannet, version 3.1.^5,6^

**Figure.**
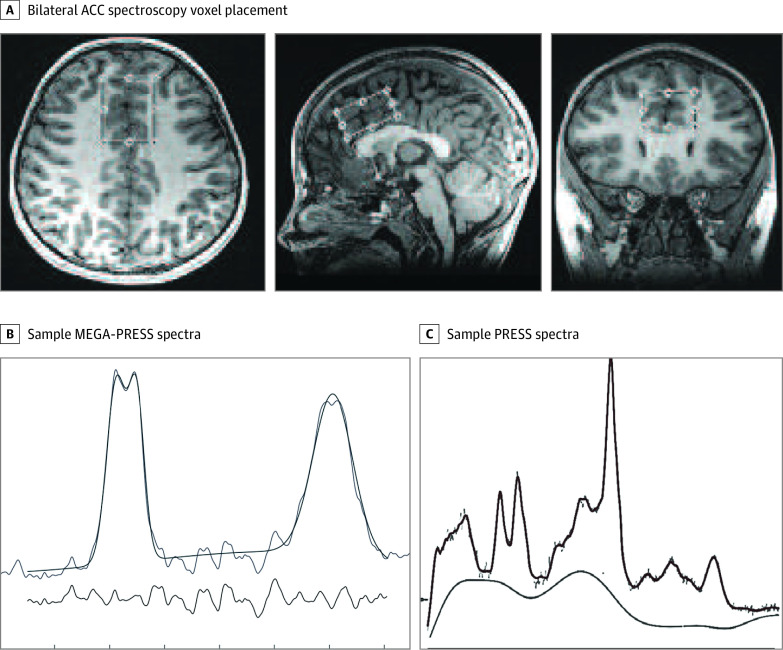
Bilateral Anterior Cingulate Cortex (ACC) Voxel Location and Example of Mescher-Garwood–Point-Resolved Spectroscopy (MEGA-PRESS) and PRESS Spectra A, bilateral ACC spectroscopy voxel placement (20 × 30 × 40 mm) over an anatomical brain scan (T1-weighted image) in multiple planes. B, Sample MEGA-PRESS spectra obtained from the bilateral ACC. C, PRESS spectra obtained from the bilateral ACC.

Data were inspected for missing values and to meet the assumptions for parametric analysis (SPSS Statistics, version 25; IBM Corporation). Because this is the first study, to our knowledge, of this nature, a sample size of convenience was used. Independent sample *t* tests were used to determine group differences (1-sided in keeping with our directional hypotheses). Cohen *d* values were reported to indicate the effect size for potential differences between groups. Bonferroni corrections were used to correct for multiple comparisons (set at a statitical significance threshold of *P* = .02). We followed the Strengthening the Reporting of Observational Studies in Epidemiology (STROBE) reporting guideline.

## Results

Of 26 children with ADHD, 13 were male and 13 were female, with a (SD) mean age of 11.6 (2.5) years; of 25 TDC participants, 13 were male and 12 were female, with a (SD) mean age of 11.2 (2.8) years (χ^2^ = 0.02; *P* = .86). We found no differences in GABA (*t*_48_ = 0.49; *P* = .63; Cohen *d* = 0.13), glutamate (*t*_49_ = 0.17, *P* = .87; Cohen *d* = 0.13), or Glx (*t*_49_ = 0.61; *P* = .55; Cohen *d* = 0.16) concentrations in the bilateral ACC voxel (Table). Participants tolerated the 9-minute MEGA-PRESS and 4-minute PRESS sequences with high-quality spectral fit based on visual inspection and the Cramer-Rao lower-bound threshold (<20%).

**Table.** Demographic and Behavioral Characteristics of Participants

Characteristic	Children with ADHD (n = 26)	TDC participants (n = 25)	*t* Value	Cohen *d* value	*P* value
Age, mean (SD), y	11.61 (2.53)	11.22 (2.75)	0.53	0.15	.51
Mean (SD) Conner-3 T-score^a^					
Inattention	75.69 (11.40)	55.56 (8.97)	7.04	1.96	<.001
Hyperactivity	78.81 (11.67)	53.81 (8.23)	8.88	2.48	<.001
Mean (SD) GABA concentration, mM	2.45 (0.34)	2.49 (0.26)	–0.49	0.13	.63
Mean (SD) glutamate concentration, mM	9.37 (1.29)	9.43 (1.06)	–0.17	0.13	.87
Mean (SD) Glx, mM	11.59 (3.11)	11.18 (1.43)	0.60	0.17	.55

Abbreviations: ADHD, attention-deficit/hyperactivity disorder; Conner-3, third edition of Conners manual that assesses cognitive, behavioral, and emotions problems; GABA, γ-aminobutyric acid; Glx, glutamine and glutamate; TDC, typically developing control.

^a^ The mean (SD) T-score on the Conners-3 rating scale is 50 (10). T-scores higher than 70 indicate clinically significant concerns.

## Discussion

This study is the first, to our knowledge, to investigate the association of GABA and glutamate concentration in children with ADHD. We found no differences concentration between children with ADHD and TDC participants. We demonstrated feasibility to collect high-quality GABA data from unmedicated children with ADHD. Although the results contrast with our hypotheses, the null findings add valuable information to the current magnetic resonance spectroscopy literature. Our main limitation was the size and composition of the voxels and does not rule out glutamate or GABAergic differences in subregions. Furthermore, to our knowledge, a limited number of studies have investigated the GABA concentration in children with ADHD, with inconclusive findings.^3^ Our approach can be replicated in other brain regions, adding critical data to our understanding of the underlying neurobiology of pediatric ADHD, which can allow for the development of targeted treatments and potential biomarkers for ADHD.
